# Effect of Dual Shot Peening on Microstructure and Wear Performance of CNT/Al-Cu-Mg Composites

**DOI:** 10.3390/ma17205066

**Published:** 2024-10-17

**Authors:** Wenlong Zhu, Huabing Liu, Shilong Xing, Chuanhai Jiang, Vincent Ji

**Affiliations:** 1 School of Materials Science and Engineering, Shanghai Jiao Tong University, Shanghai 200240, China; 2 Green & Smart River-Sea-Going Ship, Cruise and Yacht Research Center, Wuhan University of Technology, Wuhan 430062, China; 3 Institut de Chimie Moléculaire et des Matériaux d’Orsay, Université Paris-Saclay, 91405 Paris, France

**Keywords:** shot peening, CNT/Al-Cu-Mg composites, microstructure, residual stress, hard second phases, wear performance

## Abstract

This work systematically investigated the effect of dual shot peening (DSP) and conventional shot peening (CSP) on the microstructure, residual stress and wear performance of the CNT/Al-Cu-Mg composites. The results indicated that compared with CSP, DSP effectively reduced surface roughness (Rz) from 31.30 to 12.04 μm. In parallel, DSP introduced a smaller domain size (33.1 nm) and more dislocations, higher levels compressive residual stress and a stiffer deformation layer with deeper affected zones. Moreover, DSP effectively improved the uniformity of the surface layer’s microstructure and residual stress distribution. The improvement is mainly due to secondary impact deformation by microshots and fine grain strengthening. In addition, the transformation of the hard second phases such as Al_4_C_3_ and CNT and its effects on improving the surface strength and deformation uniformity were discussed. Significantly, DSP improved the wear resistance by 31.8% under the load of 6 N, which is attributed to the synergistic influence of factors including hardness, compressive residual stress, surface roughness, and grain size. In summary, it can be concluded that DSP is an effective strategy to promote the surface layer characteristics for CNT/Al-Cu-Mg composites.

## 1. Introduction

Aluminum matrix composites (AMCs) have gained much attention and extensive applications in the aerospace, automobile and other mechanical industries due to their lightweight, high ductility, and good toughness [1]. As new types of AMCs, CNT/Al composites inherit their advantages. Meanwhile, the integration of reinforcement carbon nanotube (CNT) further increases the strength [2,3] and hardness [4]. After years of research, CNT/Al composites exhibit promising properties as viable materials for the automotive, power, and aerospace industries [5,6], specifically in gear, bearings, high-speed train samples, and other parts [7,8]. However, in practical applications, these components are often subject to wear damage and fatigue damage due to uneven cyclic stress and friction damage. Thus, enhancing the friction performance can notably prolong service life, elevate overall stability, and decrease energy consumption in transmission components, ultimately resulting in improved operational efficiency. Considering that most failures occur on the component surface [9], grain refinement based on surface mechanical treatment is an effective strategy to solve this challenge.

Shot peening (SP) is one of the most widely used surface treatment techniques due to its easy control and high efficiency. SP can introduce a suitable compressive residual stress (CRS) field, thus inhibiting crack initiation and propagation. On the other hand, SP can refine the surface layer microstructure, leading to notable improvements in hardness [10] and wear resistance [11]. Up to now, SP has been shown to be effective in prolonging service life and enhancing the durability of CNT/Al composites [12,13]. However, the improvement of the service life of components is limited by conventional shot peening (CSP) since a higher SP intensity and coverage could increase surface roughness [14], microcracks [15], and even work-softening [16]. As a result, some novel SP techniques, such as microshot peening, warm SP, and stress SP, have come up with.

Microshot peening, a novel form of SP technology, has emerged in recent years [17]. This innovative technique involves impacting the component surface with shots smaller than 0.2 mm in diameter. Compared to CSP, microshot peening uses shots in a very small size and impacts the material at velocities two to three times higher [18]. This advanced method offers several advantages, including the ability to introduce larger CRS, increase the surface strength, create a gradient structure, reduce surface roughness, achieve higher levels of surface uniformity, and even produce fine-textured nanoscale surface microstructure [19]. Nevertheless, compared to that of CSP, the affected depth of the deformation layer induced by microshot peening is smaller. As a result, it is necessary to combine the advantages of CSP and microshot peening to improve the surface properties of components. Nowadays, some researchers utilize the dual shot peening (DSP) technique to improve the surface characteristics of different alloys further. However, there is a lack of adequate research on combining microshot peening with CSP to improve component performance of CNT/Al composites.

This work aims to take advantage of the combination of conventional shot peening and microshot peening to improve the surface characteristics of CNT/Al-Cu-Mg composites. The surface layer microstructure, CRS field, and wear performance were comprehensively analyzed. The microstructure was quantitatively evaluated by the Rietveld fitting method and observed by TEM. The residual stress (RS) distribution was measured by the XRD method. Microhardness and wear performance were used to evaluate the mechanical property. The underlying mechanisms responsible for the improvement were discussed. The research result could provide some good insights for the application of DSP on the CNT/Al-Cu-Mg composites and other alloys.

## 2. Experimental Method

### 2.1. Material Preparation

The CNT/Al-Cu-Mg composites were prepared by the powder metallurgy method, and the chemical compositions are shown in Table 1. The materials were prepared by metal powders using shift-speed ball milling at 135 rpm for 8 h and 270 rpm for 1 h. This was followed by cold pressing at 500 MPa, sintering at 570 °C for 6 h, hot extrusion at 450 °C, solution treatment at 530 °C for 3 h, quenching in water, and ageing treatment at 130 °C for 24 h [20]. The resultant material was then cut into strip samples of 60 × 12 × 3 mm by wire cutting and polished with 1000 mesh silicon carbide before SP to eliminate the processing residual stress.

**Figure 1:**
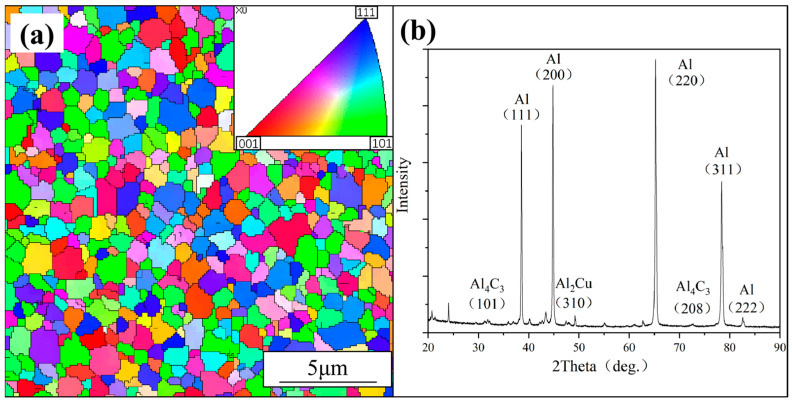
Microstructure of the original material (**a**) the EBSD characterization (**b**) the XRD pattern.

Figure 1a is the EBSD inverse pole figure map (EBSD Mira3, Czech Republic) of the original material. It shows that the primary grain size is approximately 1 μm in this plane. Figure 1b shows the XRD pattern (XRD Ultima IV, Japan) of the original material, indicating the composite consists of abundant Al phase and minor Al_4_C_3_ and Al_2_Cu phases. The presence of Al_4_C_3_ and Al_2_Cu can be attributed to the reaction between CNTs, Cu particles, and Al particles during the shift-speed ball milling [21] and sintering process [22,23]. It is worth noting that the content of CNTs is less than 1.5 wt% after the formation of the Al_4_C_3_ phase, which is below the detection limit of XRD [24]. Therefore, no carbon diffraction peaks were detected in this composite.

### 2.2. Shot Peening and Friction Experiment

The samples of CNT/Al-Cu-Mg composites contain DSP samples, CSP samples, and unpeened counterparts. SP was performed using an air blast machine. Specifically, the CSP medium used was the S110 steel shot (diameter 0.30 mm), while the DSP medium consisted of the S110 steel shot and Z150 ceramic microshot (diameter 0.18 mm). The peening intensity was determined by measuring the arc height of the A-type arm specimen in millimeters [25]. The diameter of the peening nozzle was 15 mm, which was positioned 100 mm from the sample surface. The test sample achieved a peening coverage of 100%, and the key parameters are shown in Table 2.

A reciprocating friction wear tester (UMT-Tribolab, BRUKER, USA) was used to conduct dry sliding wear tests on each specimen at room temperature. Each test was repeated three times. GCr15 steel balls (with a diameter of 10 mm) were used for the wear parts. The wear measurements were carried out under 2, 4, and 6 N applied loads, and the frequency was 5 Hz. The scratch length was 6 mm and the wear times were all 10 min.

### 2.3. Characterization of Surface Layer Characteristics

The surface morphology of the peened samples was examined using a scanning electron microscope (SEM, VEGA3 XMU, Czech Republic) operating at 15 KV. The 3D morphology and roughness of the samples were evaluated using a stylus profiler (DektakXT, USA). The microstructure of the samples was observed by a field emission transmission electron microscope (TEM) (Talos F200X G2, USA) specifically designed for material characterization. TEM samples were prepared by cutting from the surface deformation layer of the sample and then thinned by ion milling in a Gatan PIPS machine (695, USA). RS was analyzed using an X-ray stress analyzer (LXRD, Proto, Canada) using the sin^2^ψ method [26]. Cu Kα radiation with a wavelength of λ = 2.291 Å was used to detect Al (311) diffraction peaks. In addition, the hardness distribution along the depth was measured using a digital microhardness tester (DHV-1000, Beijing, China) with a loading weight of 0.98 N and a dwell time of 15 s. The hardness value of each layer was measured five times, and the average value was calculated as the final result. To obtain the RS, XRD patterns, and hardness at different depths, a continuous material removal process from the top surface to the substrate was performed by electrochemical polishing [27]. The electrochemical removal was performed in a saturated NaCl solution with a direct current of 3 A/D^2^.

Analysis of the microstructure within the deformation layer was performed using the Rietveld refinement method [28] in the highly regarded academic software MAUD 2.71. The complete XRD patterns were carefully fitted, accounting for instrumental factors and optical diffraction effects. The Rietveld refinement made extensive use of the widely used pseudo-Voigt (pV) function [29] to accurately fit the peak shape of Kα1 and Kα2. The mathematical expression for the peak profile can be expressed as follows:

(1) $$V\left(2\partial \right)=\sum_{\alpha 1\alpha 2}I_{nt}(1-\eta )(1+S^{2}{)}^{-1}+\eta exp(-ln2\times S^{2})$$

where S = (2*∂* − 2∂_0_)/β, ∂_0_ was the Bragg angle of Kα1 radiation. Int, η, and β were the scale parameters of the pV function, the Gaussian component, and the full width at half maximum, respectively.

The POPA model [30] was used to elucidate the domain size and microstrain of the CNT/Al-Cu-Mg composites. The Warren model was used to modify the analysis of the displacement, broadening, and asymmetry observed in the profile due to deformation or twin faults. Using the domain size and microstrain obtained by Rietveld refinement, the dislocation densities can be quantified using the following equation:

(2) $$\rho =\frac{2\sqrt{3}}{b}\frac{\langle \varepsilon^{2}\rangle^{\frac{1}{2}}}{D}$$

where *ρ*, ε, D, and b represent the dislocation density, microstrain, domain dimension, and Burgers vector, respectively. 〈ε^2^〉 denotes the weighted average of ε^2^ after multiple measurements. The use of X-ray diffraction data for dislocation density analysis has distinct advantages over other methods. In particular, this method allows statistical analysis of dislocation density in millions of grains [31].

## 3. Results and Discussion

### 3.1. Surface Morphology

**Figure 2:**
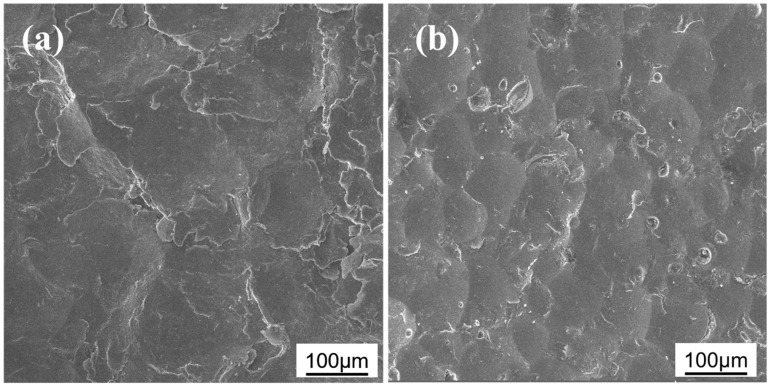
SEM images of samples’ surface morphology (**a**) after CSP (**b**) after DSP.

Figure 2 depicts the surface morphology of the sample after the processes of CSP and DSP. After CSP, the sample surface presents wrinkles and pits caused by dramatic plastic deformation. In addition, the pits left on the surface exceed 100 μm in diameter and even a limited number of cracks are present at the SP overlap zone, indicating that the alloy has exceeded its plastic deformation limit. Conversely, after DSP, the integrity of the surface is significantly improved, with a noticeable reduction in the number of wrinkles and the diameters of pits. Furthermore, there are no microcracks on the surface. This variation is attributed to the smaller micro-ceramic shots in the DSP process, which ensures a more evenly distributed impact on the surface. As a result, the surface deformation becomes more uniform, resulting in an improvement in the structural integrity of the surface.

**Figure 3:**
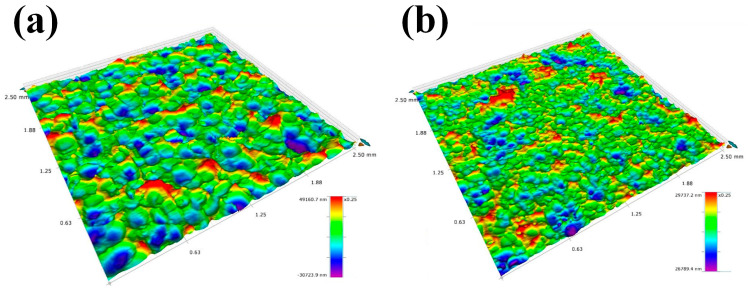
3D surface topographies of samples (**a**) after CSP (**b**) after DSP.

In addition, the 3D surface topographies of the two kinds of samples are shown in Figure 3a,b, respectively. Notably, CSP and DSP both bring about rough surfaces with numerous peaks and dents. Compared to the CSP sample shown in Figure 3a, the dents on the DSP sample, shown in Figure 3b, are smaller and denser. The detailed surface roughness values were obtained in Table 3. After CSP, the surface roughness values, Ra and Rz, are 4.76 and 31 μm, respectively. By applying the DSP technique, the surface roughness values, Ra and Rz, decreased to 2.53 μm and 12 μm, respectively. The Rz value was significantly reduced by 61.5%. It suggests that micro-ceramic shots weakened the height of the extruded ridges induced by CSP. It is widely accepted that the increase in surface roughness will enlarge the stress concentration factor and accelerate the initiation of fatigue sources to the surface of the material [32], thus deteriorating the fatigue properties, corrosion resistance, and corrosion fatigue. Consequently, in most cases, the control of surface roughness of shot-peened components is an effective strategy to prolong their service life in industry.

### 3.2. X-ray Analysis

**Figure 4:**
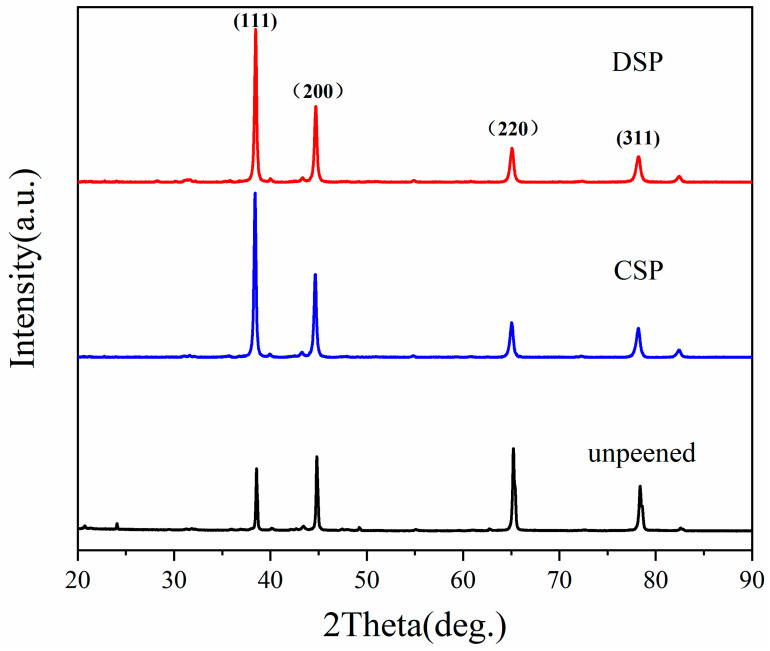
XRD patterns of each treated sample at the top surface.

The XRD patterns at the top surface layer of the samples before and after SP are presented in Figure 4. It is observed that no new diffraction peak appears, and no phase transformation occurs in CNT/Al-Cu-Mg composites during the SP process. In parallel, SP leads to the broadening of the half-width of Al (111) diffraction peak, which is due to the grain refinement and lattice distortion. The refinement of the domain size contributes to the enhancement of the strength of the surface. Moreover, the relative intensity of each peak varies significantly before and after SP, which is due to the variation of fiber textures. During the fabrication of CNT/Al-Cu-Mg composites, there are preferred orientations along the (220) and (311) crystal plane directions. After CSP and DSP, the impact of numerous shots on the sample surface results in a large number of dislocations accumulating and more sub-grain/grain boundaries. This brings about a rotation of grain orientations and the disruption of the original texture. The disruption of the original texture improves the uniformity of the surface properties in different crystal plane orientations.

**Figure 5:**
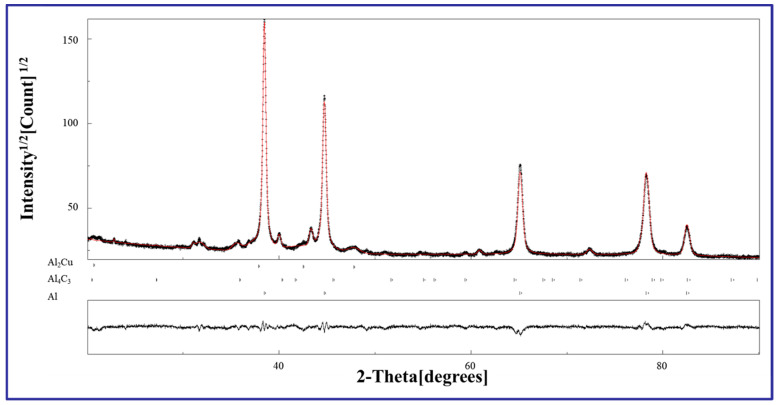
Fitting results of XRD patterns of peened samples (DSP).

To obtain further microstructure information about the deformation layer of CNT/Al-Cu-Mg composites, the Rietveld fitting of each sample was performed by MAUD software. Figure 5 displays the fitting curve of the DSP sample at the top surface. It can be seen that the fitting curve and the measured curve are highly consistent. For Rietveld refinement, the Sigma (Rwp/Rexp) value is desirable to be controlled at a level below 2 [33]. In this work, the Sigma value after each Rietveld refinement is 1.62, indicating the rationality of the fitting results.

**Figure 6:**
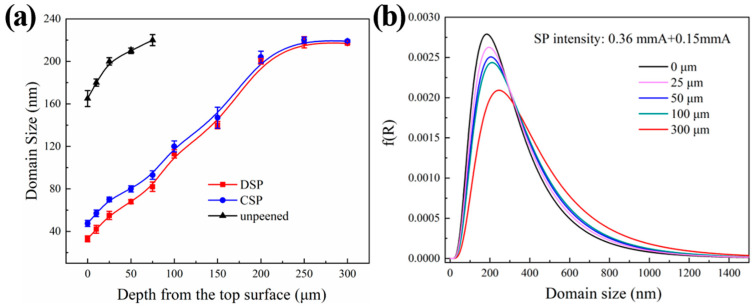
Distributions of different depths of domain size from the fitting result of Al (111) planes: (**a**) average domain size; (**b**) the probability.

According to the Rietveld refinement method, the depth distributions of domain size are obtained. Figure 6a shows a comparison of domain size at different depths. Compared to the unpeened counterpart, domain sizes at the top surface after CSP and DSP decreased to 47.5 nm and 33.1 nm, respectively. The variation trend of domain size of the two samples is similar. As the depth increased, the domain size gradually increased and reached a stable value of approximately 220 nm after the depth reached 250 μm. The unpeened counterpart showed a slight reduction in surface domain size to 165 nm compared to the matrix, due to the introduction of slight plastic deformation during the preparation process. Nevertheless, this affected zone was shallow, with a depth of less than 100 μm. Figure 6b shows the logarithmic distribution of domain size with layer depth after DSP. The figure shows that surface deformation was most intense, and grain refinement was most pronounced. The domain size distribution range was narrow, indicating a uniform sample microstructure with the highest probability of occurrence observed at approximately 180 nm. As the layer depth increased, the domain size distribution range widened, and the maximum probability domain size value increased to 215 nm at a depth of 300 μm. The result shows that the domain size exhibited significant gradient variations with layer depth change, and the effect of DSP on reducing domain size was evident.

**Figure 7:**
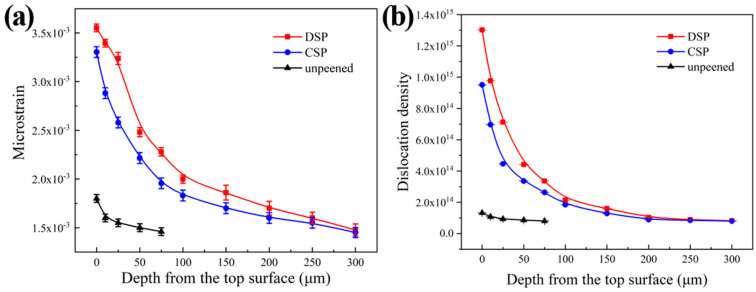
Distributions along the depth from the fitting result of Al (111) planes (**a**) microstrain (**b**) dislocation density.

Figure 7 shows the distribution of microstrain and dislocation densities at different depths of CNT/Al-Cu-Mg composites. It shows that the variation trend of microstrain and dislocation density are similar and contrary to that of domain size. Specifically, they gradually decrease as the layer depth increases, and the rate of decrease shows a gradient change along the layer depth. At the top surface, the microstrain and dislocation densities are 3.55 × 10^−3^ and 1.30 × 10^15^ m^−2^ after DSP, while they are 3.30 × 10^−3^ and 9.51 × 10^14^ m^−2^ after CSP. In the upper 50 μm, both microstrain and dislocation density decreases rapidly with increasing depth. Beyond 50 μm, the decrease rate becomes slower, finally reaching a stable level at the matrix depth after 250 μm. This behavior can be attributed to the fact that microstrain and dislocation densities are primarily influenced by the amount of plastic deformation in the material. The application of SP induces significant plastic deformation on the surface of the sample, resulting in dislocation multiplication and accumulation at grain boundaries. In addition, the gradient change of plastic deformation along the depth direction causes the gradient change of microstrain and dislocation density. The DSP further enhances the plastic deformation of the surface and subsurface of the sample by transferring more kinetic energy upon impact. This results in an increased level of microscopic stress in the shot-peened layer, leading to a further increase in dislocation density and a more pronounced strengthening effect.

**Figure 8:**
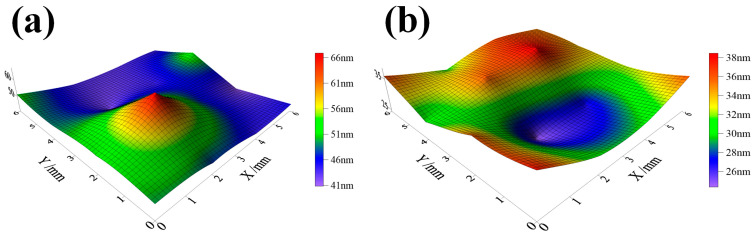
The domain size distribution of samples’ top surfaces (**a**) after CSP (**b**) after DSP.

In order to study the uniformity of surface structure, the domain size distribution was determined through XRD measurements at 1.0 mm intervals and within the 4 × 4 mm area on the sample top surface, as shown in Figure 8. It can be seen that after CSP, the domain size ranges from 41 nm to 66 nm, while after DSP, it ranges from 26 nm to 38 nm. Additionally, the average domain size is significantly reduced from 47.5 nm after CSP to 33.1 nm after DSP. The corresponding standard deviation is 6.1 nm to 3.9 nm. This reduction represents a refinement in grain size, which promotes deformation over a greater number of grains. In addition, the reduced standard deviation and range indicate a more uniform structure, resulting in improved surface integrity and the impact-resisting properties of CNT/Al-Cu-Mg composites.

The substantial grain refinement after DSP can primarily be attributed to the enhanced impact deformation of the surface by microshot peening. During the initial stages of plastic deformation, large grain deformation was preferred, with “soft orientation” and “soft grains”. For Al composite, if the impact is close to <110> lattice cells’ orientation, these grains are more susceptible to deformation. The grains in the soft orientation were limitedly deformed when they were impacted and were divided by dislocations in different directions to form sub-grains. With further strain, the lattice cells orientation in large grains in the non-deformable direction gradually rotated and finally changed to the easily deformed direction. The large grains continued to deform during the subsequent impact. The microshot peening process involves numerous small shots impacting the surface, ensuring that the remaining large grains that were not fully deformed in the less-deformed regions during CSP, undergo further impact and deformation into smaller grains. This results in an improvement in microstructure uniformity. At the same time, second phases, such as Al_4_C_3_ and CNT, were also evenly distributed in the material in the microshot peening, which further limited the dislocations move and the growth of grains.

### 3.3. Effect of the Second Phase

As the second phase with the highest content in the alloy, the Al_4_C_3_ phase plays a vital role in the strengthening mechanism of the material. Therefore, the structure changes of the Al_4_C_3_ phase before and after shot peening were investigated by means of XRD. The Al_4_C_3_ phase, formed during the material preparation process, is distributed near the grain boundary of the aluminum matrix [34]. As a hard phase, it requires high shear stress for dislocation bypassing, thereby impeding dislocation movement and increasing the limit for dislocation accumulation. This dispersion strengthening, via the Orowan mechanism [35], allows the surface of the material to withstand greater impact loading. The shear stress τ required for the dislocation line to bypass the second phase is given by:

(3) $$\tau =\frac{0.13Gb}{\lambda }ln\frac{r}{b}$$

where G, λ, and r represent the elastic modulus, the interparticle distance, and the size of Al_4_C_3_.

**Figure 9:**
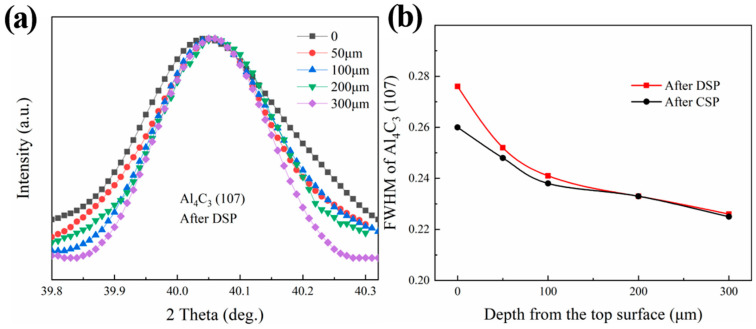
Changes of Al_4_C_3_ (107) diffraction peak along the depth (**a**) peak shape after DSP (**b**) FWHM after CSP and DSP.

Figure 9 shows the change in full width at half maximum (FWHM) of the Al_4_C_3_ (107) diffraction peak at different depths after the DSP and CSP. The FWHM serves, to some extent, as an indicator of the corresponding domain size change. The results show a slight broadening of the FWHM of the Al_4_C_3_ (107) diffraction peak. Although the change in value is minimal, the change in the diffraction peak patterns is evident after the DSP (as shown in Figure 9a). This small change can be attributed to the fact that the Al_4_C_3_ phase is a type of hard particle with a high Young’s modulus compared to the aluminum matrix, where k = P/(−dV/V). Consequently, the Al_4_C_3_ phase presents minimal change when subjected to the same impact force. The diffraction peaks of the Al_4_C_3_ phase broadening indicate an increase in the number of crystal planes participating in coherent diffraction and alterations in the structure and distribution of the Al_4_C_3_ phase. After distorting the Al_4_C_3_ phase and experiencing a significant number of dislocation movements, the grain size is refined, and the distribution becomes more uniform, further hindering the plastic deformation of the material. As shown in Figure 9b, compared to CSP, the FWHM of the Al_4_C_3_ broadens more obviously in the surface layer after DSP, indicating more changes in the structure of Al_4_C_3_. Additionally, in addition to the direct impact at the top surface Al_4_C_3_, the subsurface Al_4_C_3_ primarily serves to transmit force within the matrix Al phase, resulting in minimal changes in the subsurface.

The CNTs distributed at the grain boundary also have a high Young’s modulus due to their nanoscale geometric properties, which hinder dislocation movement. The strengthening mechanism [36] of the CNTs on the surface of the material is similar to that of Al_4_C_3_ but with a more dispersed distribution. Additionally, the growth of aluminum grains is restricted by CNTs, resulting in no coarse grains in CNT/Al-Cu-Mg composites.

### 3.4. TEM Observation

**Figure 10:**
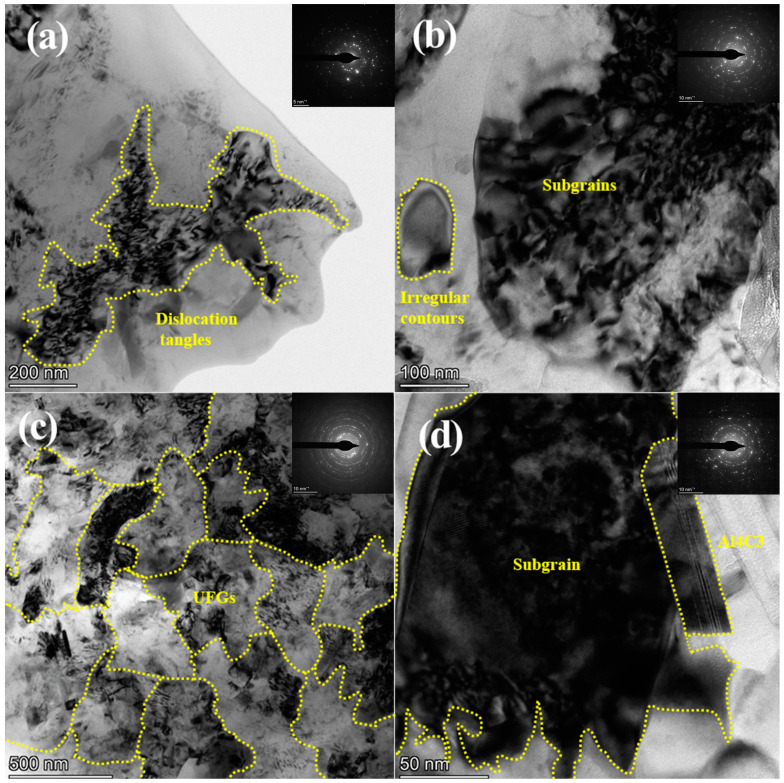
TEM micrographs and SAED of peened samples (CSP) (**a**) tangle of dislocations (**b**) dislocation cells (**c**) dislocations and ultrafine grains (**d**) second phase next to the dislocation cell.

In order to directly investigate the microscopic changes of the microstructure after SP, the surface layer of the DSP sample was observed by TEM. Figure 10 shows the TEM bright field image with different degrees of deformation and the corresponding selected area electron diffraction (SAED) pattern. It seems that the surface layer undergoes dramatic plastic deformation after DSP. As shown in Figure 10a, the dislocation tangles divide the whole grain into smaller substructures. The corresponding diffraction spots become diffraction rings, as shown in the SAED pattern, indicating the refinement of original coarse grains (1 µm). Figure 10b presents the finer microstructure with subgrains and clearer diffraction rings. With further increasing strain, as shown in Figure 10c, ultrafine grains with a mean grain size of nearly 500 nm are formed, and the integral diffraction rings can be detected. Figure 10d shows a subgrain formed in the vicinity of Al_4_C_3_, due to significant accumulation of dislocations. As mentioned earlier, Al_4_C_3_, which acts as a harder second phase, remains pinned within the matrix with minimal deformation, requiring the bypassing of dislocations around the Al_4_C_3_ phase to maintain their motion. The presence of Al_4_C_3_ impedes dislocation movement and plays a role in dispersion strengthening.

At the initial stage of plastic deformation, the atomic misalignment in the grains leads to the formation of a large number of dislocations. During the deformation process, both new dislocations are created, and existing dislocations continuously slip. The matrix material is Al with a face-centered cubic structure that has multiple slip systems. Dislocations with different slip directions interact, leading to the formation of dislocation tangles. In addition, the SAED pattern shows discontinuous diffraction spots. In regions of more severe deformation, dislocations continue to accumulate at grain boundaries and sub-grain boundaries, leading to the formation of dislocation cells. In addition, irregular extinction contours and the presence of ill-defined transition zones between grains and grain boundaries indicate the high-energy unstable nature of the microstructure and the elevated state of residual stress [37,38]. With the continuous intensification of plastic deformation, the substructure is enveloped by various dislocations and further divided into smaller subgrains and grains.

It is important to note that small deviations in crystal orientation, called domains, similar to dislocation cells and subgrains [39], can be seen in the X-ray diffraction peak. In TEM, however, grains only with high-angle grain boundaries can be seen. Consequently, the grain size observed in TEM appears larger than the domain size calculated by the Rietveld fitting method.

### 3.5. Residual Stress and Hardness Distributions

**Figure 11:**
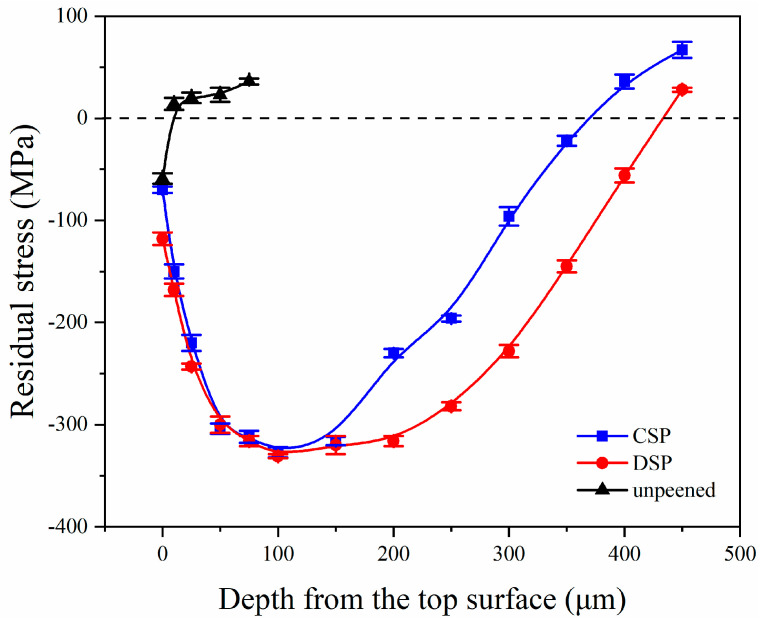
Changes of residual stress along the depth.

Figure 11 shows the residual stress distribution at the surface layer of each sample before and after SP. It can be seen that the unpeened counterparts have a small amount of CRS on the surface of −59 MPa. As mentioned, this is introduced during the fabrication process. However, the depth of the layer affected by this stress is less than 25 μm, which is insufficient to produce a strengthening effect. After CSP and DSP treatments, a satisfactory CRS field was introduced at the deformation layer. After CSP, the magnitude of surface CRS reaches −70 MPa. This RS initially increases and then decreases with increasing depth. In particular, at a depth of 100 μm, the maximum CRS reaches −316 MPa, with a range of influence exceeding. Moreover, the affected depth exceeds 350 μm. For the DSP sample, the CRS value increases to −118 MPa at the surface and reaches a maximum value of −320 MPa at 100 μm. Compared with CSP, the maximum CRS remains nearly unchanged after DSP. This is attributed to the high SP intensity of CSP, which brings the maximum CRS close to the yield point of the original material. While the surface grain refinement and the introduction of more plastic deformation lead to the increase of surface CRS. It is worth noting that the effect of DSP on the CRS field is mainly embodied in the magnitude near the surface and the affected depth over 400 μm. In conclusion, DSP induces a higher CRS field at the sample surface, resulting in a superior strengthening effect on the surface of the material.

**Figure 12:**
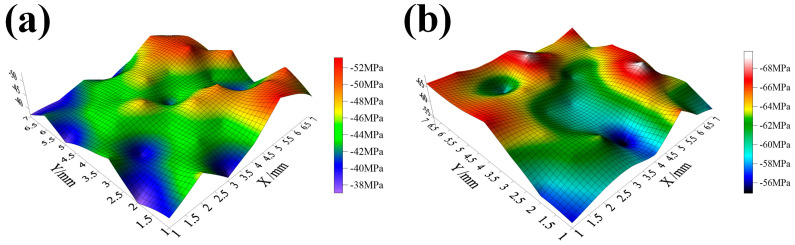
The residual stress distribution of samples’ top surfaces (**a**) after CSP (**b**) after DSP.

In order to study the distribution of CRS, the CRS value was measured at 1.0 mm intervals, and the distribution of CRS within the 7 × 7 mm area at the sample surface was evaluated. In Figure 12, it shows the magnitudes of CRS after CSP and DSP.

After the application of CSP and DSP, both methods resulted in CRS on the surface, but with different magnitudes. It shows that after DSP, the average value of CRS on the sample surface increased from −45 MPa to −63 MPa, while the standard deviation decreased from 4.3 MPa to 3.5 MPa. The increase in CRS contributes significantly to the improvement in fatigue resistance, accompanied by a reduction in both standard deviation and range. These results indicate that the surface plastic deformation becomes more uniform, and its overall performance becomes more stable after DSP. The improvement of CRS uniformity is primarily attributed to three aspects: Firstly, the surface continues to undergo plastic deformation, which makes the deformation degree of the microstructure in the affected layer more uniform. Secondly, DSP endows higher peening coverage than CSP, with a smaller diameter and a greater number of miroshots, resulting in a more dispersed impact. At the same time, as mentioned in Figure 8, the domain sizes are reduced and distributed more uniformly, demonstrating that the grains will interact with each other and more grains will coordinate deformation. Thirdly, the decrease in roughness reduces the possibility of sharp deformation zones, which also results in a narrower range of CRS distribution.

**Figure 13:**
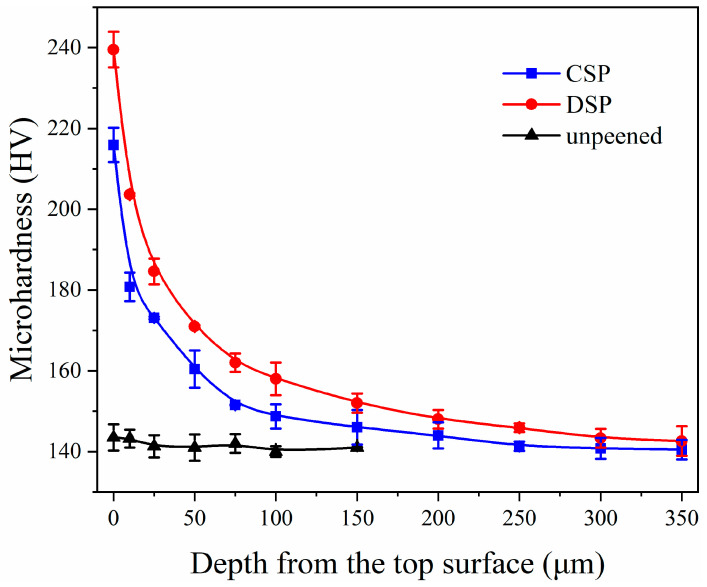
The hardness distribution of samples’ surfaces.

Hardness is an important indicator of material mechanical properties because it directly reflects the strength. The distribution of hardness with depth before and after SP is shown in Figure 13. The surface hardness of the sample without SP remains constant at around 140 HV, regardless of the plating depth. After SP treatment, surface hardness increases to 216 and 240 HV, which corresponds to CSP and DSP samples, respectively. This indicates that SP treatment significantly improves surface layer hardness. Compared with CSP, DSP induces refiner domains and more dislocations, thus generating higher surface hardness. Moreover, DSP further increases the depth of the surface deformation layer from 150 (CSP) to 200 μm.

It is worth noting that the improvement of dislocation density, CRS, and mechanical property caused by DSP on CNT/Al-Cu-Mg composites is mainly reflected on the near-surface layer (<50 μm). This is determined by the character of microshot peening. Due to the small shot size, the affected depth of microshot peening is comparatively smaller than that of CSP. In parallel, the modification induced by microshot peening is embodied in the near-surface layer, especially for the CRS factor. Therefore, by applying the microshot peening on the CSP sample surface, namely the DSP method, we obtain superior CNT/Al-Cu-Mg composites with finer surface layer microstructure, higher-level CRS and affected depth.

### 3.6. Wear Performance

**Figure 14:**
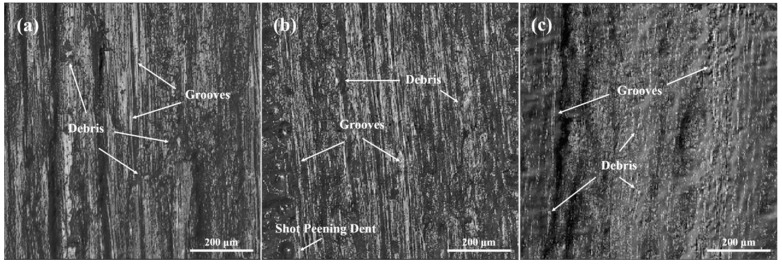
The DIC images of the worn surface under 2 N load. (**a**) unpeened (**b**) after CSP (**c**) after DSP.

The Differential Interference Contrast (DIC) images of the worn surface are presented in Figure 14 for studying the wear mechanism of CNT/Al-Cu-Mg composites. The surface of the samples showed significant ridges and grooves with inclusions of metal debris, indicating that the wear mode did not change. It was both adhesive wear before and after SP. The hard particles of the GCr15 steel balls scraped the solid surface of the friction pair, resulting in the generation of metal debris and direct loss of surface material. Some metal debris was pressed into the friction surface under the external load. In addition, no obvious peeling off region was observed in the test, indicating that sliding friction was the primary mechanism. Comparative analysis revealed that the grooves and debris were most pronounced in the unpeened samples. Shallower grooves and reduced debris volume were observed in the CSP ones, and the changes were even more pronounced in the DSP samples.

**Figure 15:**
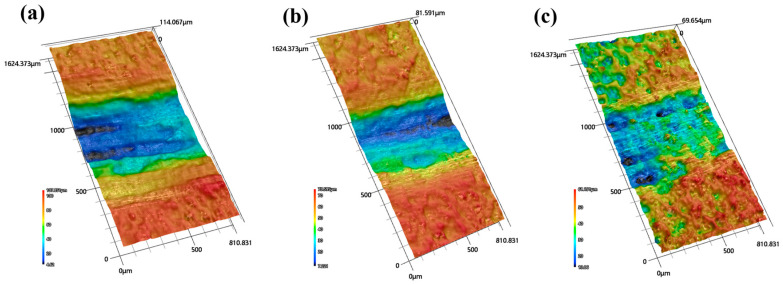
The 3D topography of the worn surfaces under 2 N load. (**a**) unpeened (**b**) after CSP (**c**) after DSP.

Figure 15 shows the 3D topography of the worn surfaces and clearly shows the overall changes in the depth of ridges before and after shot peening. The maximum depth of the ridges before shot peening was 108 μm, which decreased to 76 μm after CSP and further decreased to 61 μm after DSP.

**Figure 16:**
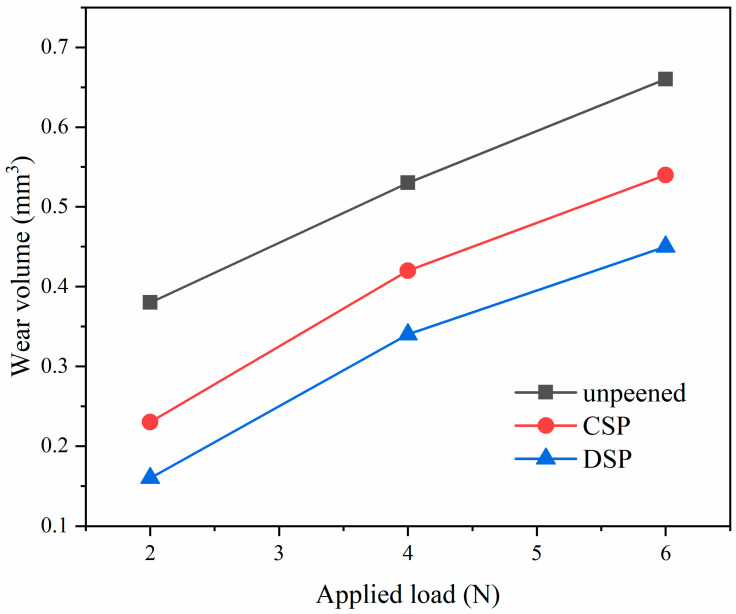
The wear volumes of the samples.

To quantify the impact of SP on wear performance, a statistical analysis of wear volumes was conducted, as depicted in Figure 16. A positive correlation was observed between the external load and the wear volume on the sample surface. Especially for unpeened samples, there is almost a linear relationship between the external load and the wear volume. Notably, CSP treatment significantly reduced the wear volume compared to unpeened samples, and DSP further diminished this volume. Specifically, at an external load of 2 N, the untreated sample exhibited a volume loss of 0.38 mm^3^, while CSP and DSP samples displayed volumes of 0.23 mm^3^ and 0.16 mm^3^, representing respective reductions of 39.5% and 57.9%. For 6 N, the volume loss for unpeened, CSP, and DSP is 0.66 mm^3^, 0.54 mm^3^, and 0.45 mm^3^, respectively, with reductions of 18.2% and 31.8% for CSP and DSP. These findings demonstrate that DSP treatment significantly enhances the wear resistance of the sample surface. The higher percentage increase in friction resistance at 2 N is due to the gradient microstructure formed by shot peening. The closer to the surface, the higher the hardness increase (as mentioned in Figure 13), and the more obvious the refinement of domain size (as mentioned in Figure 6). J.V. et al. [40] discovered that the wear rate of AA2017 alloy underwent a significant 25.8% reduction following 140 s of shot peening. Similarly, Chen et al. [41] revealed that the employment of ultrasonic shot peening (SP) resulted in a noteworthy 33.58% decrement in the wear rate of Al–Zn–Mg–Cu alloy. Furthermore, C.M. et al. [42] achieved a remarkable 44.9% reduction in the wear rate of AlSi10Mg alloy through the application of laser peening. Hence, the 57.9% reduction in wear volume observed in this study within the same timeframe is highly significant. Elucidating the underlying mechanisms of this enhancement is important.

The Archard equation [43] is often used to describe the pattern of change in the amount of wear.

(4) $$W=K\frac{F}{H}$$

where W is the wear rate, K is a parameter related to material toughness, F is the external load, and H is the material hardness. As such, for a given material, an increase in surface hardness can significantly decrease the wear rate and enhance wear resistance. Additionally, for shot-peened samples, the CRS and surface roughness emerge as crucial factors influencing wear performance [44]. CRS mitigates the effects of tensile stress, inhibiting the initiation and propagation of microcracks in the surface layer. Conversely, an increase in roughness will lead to an elevated local stress concentration, which in turn will result in plastic deformation and cracking, thereby reducing the wear resistance. Apart from above factors, Y.E. et al. [45] and Yan et al. [46] revealed that grain refinement and the formation of gradient microstructure also improve the wear resistance.

Compared with unpeened counterparts, the surface hardness of CSP samples increased by 54.3% (140 to 216 HV), the CRS reached −70 MPa, and therefore, the positive effects outweighed the adverse effects of increased roughness (31.30 μm Rz). In contrast, DSP treatment further enhanced surface hardness (240 HV) and CRS (−118 MPa), while also reducing surface roughness (12.04 μm Rz). In the meantime, the reduction in both the surface domain size and the standard deviation of CRS distribution results in a more uniform surface plastic deformation, thereby enhancing the wear performance. The synergistic effects of these factors culminated in a further reduction in wear volumes for DSP samples. In conclusion, DSP treatment exhibited the most significant improvement in wear resistance.

## 4. Conclusions

This work explored the effect of DSP on the microstructure, CRS, and the hardness of the CNT/Al-Cu-Mg composites. The main conclusions are as follows:

1. Both CSP and DSP exhibit significant modifications in surface layer characteristics. In comparison with CSP, DSP generated a superior surface layer with smaller surface roughness (2.53 μm), finer domain size (33.1 nm), severe lattice distortion (3.30 × 10^−3^), higher dislocation density (1.30 × 10^15^ m^−2^), and surface CRS (−70 MPa), thus resulting in higher hardness (240 HV).
2. DSP not only reduces surface roughness and enhances the surface quality, but also effectively improves the uniformity of surface characteristics. Compared with CSP, DSP reduces the average domain size from 47.5 to 33.0 nm, and the corresponding standard deviation from 6.1 to 3.9 nm, the average surface CRS from −45 MPa to −63 MPa, and the corresponding standard from 4.3 to 3.5 MPa. These factors also have a positive influence on enhancing wear performance.
3. Al_4_C_3_, a hard second phase, plays an important role in promoting grain refinement and enhancement of hardness during plastic deformation. Meanwhile, the refinement of Al_4_C_3_ could further promote the mechanical property of the CNT/Al-Cu-Mg composites according to dispersion strengthening.
4. Under the load of 6 N, the wear volume loss of the CSP and DSP samples is reduced by 18.2% and 31.8%. This can attribute to the synergistic influence of factors including hardness, compressive residual stress, surface roughness, and grain size.

In summary, DSP can be considered an instructive method to modify the surface properties of CNT/Al-Cu-Mg composites and other alloys.

## Figures and Tables

**Table 1:** The composition of the samples.

Element	Al	Cu	CNT	Mg
Percentage (wt%)	93.5	4.0	1.5	1.0

**Table 2:** The main shot peening parameters of the samples.

Sample	Shot Material	Shot Diameter (mm)	SP Intensity (mmA)	SP Coverage (%)
CSP	Cast steel	0.3	0.36	100
DSP	Cast steel, ZrO_2_	0.3, 0.18	0.36 + 0.14	200

**Table 3:** Surface roughness values of samples.

Sample	Ra/μm	Rz/μm
CSP	4.76	31.30
DSP	2.53	12.04

## Data Availability

The original contributions presented in the study are included in the article, further inquiries can be directed to the corresponding author.
